# Recovery and Detection of Enteric Viruses from Non-Traditional Irrigation Water Sources

**DOI:** 10.3390/mps2030055

**Published:** 2019-06-30

**Authors:** Brienna L. Anderson-Coughlin, Kalmia E. Kniel

**Affiliations:** Department of Animal and Food Sciences, University of Delaware, Newark, DE 19716, USA

**Keywords:** enteric viruses, water, RT-qPCR, detection, irrigation, food safety

## Abstract

The variability of environmental water samples impacts the allowance of one method to be universally ideal for all water types and volumes. Surface and reclaimed waters can be used for crop irrigation and may be referred to as non-traditional irrigation waters as these water types may be associated with a higher risk of microbial contamination compared to groundwater. These waters are typically more microbially and chemically complex than groundwater and have a higher risk of viral contamination. To detect viruses in these water types, an infinite number of variations can be made to traditional recovery methods. This protocol was developed based on a commonly used virus adsorption and elution (VIRADEL) method. Additional steps were included to simplify and efficiently reduce particulates in the viral concentrate and remove DNA from eluted nucleic acids prior to detection. Method alterations allow for volumes up to 40 liters to be processed with consistent recovery of enteric viruses including Aichi virus, hepatitis A virus, and noroviruses belonging to genogroups GI and GII. No inhibition was observed among either surface or reclaimed water samples. This protocol could be utilized in the monitoring of a wide array of irrigation water sources throughout irrigation processes.

## 1. Introduction

Non-traditional irrigation waters can include various types of surface and reclaimed waters and are becoming more commonly used across the United States to save energy and reduce demands placed on groundwater resources [1]. Recovering and detecting viruses in surface and reclaimed waters is necessary to determine the potential risks; however, the high variability and complexity of these waters create challenges in the development of a feasible and consistent method. The U.S. Environmental Protection Agency (EPA) reports that virus adsorption–elution (VIRADEL) methods were first developed in the 1970’s when the WHO began its virological investigation of water [2]. VIRADEL methods generally involve passing water through a filter where viruses may attach. Following the initial attachment, viruses are eluted, or removed, from the filter using a solution which can be combined with mechanical disruption methods. For detection using molecular methods and infectivity assays, the volume of the eluate is typically reduced by concentration via precipitation or ultrafiltration. Over the past four decades, there have been hundreds of articles published that employ variations of virus adsorption–elution techniques. Required sample volumes, physiochemical characteristics of the water, virus concentration and numerous other variables impact the recovery of viruses. Extensive research has been explored and compiled to create this protocol and the justifications for choosing the filtration, elution, concentration, and extraction methodologies used are provided.

The efficiency of the initial filtration step of VIRADEL methods greatly impacts the overall recovery of viruses. The first VIRADEL method utilized epoxy fiber membrane and fiber-glass cartridge filters [2]. In 1983, Goyal and Gerba first published their research in which they used a VIRADEL method to detect rotaviruses in seawater [3]. After the adoption of ultrafiltration to concentrate viruses, the Virosorb 1MDS positively charged filter was created and quickly became the preferred, though costly, method of choice [2]. In 1989, research investigating the 1MDS filter was published and deemed it to be an “improved method” for efficiently recovering viruses from large volumes of water [4]. The NanoCeram positively charged filter was developed almost thirty years later in 2009 as an alternative to the traditional 1MDS filter [2]. These two electropositive filtration methods for virus adsorption are widely used today to recover viruses from treated wastewater. NanoCeram and 1 MDS filters were compared and the NanoCeram method provided greater recovery from large sample volumes, decreased inhibition downstream in PCR detection, and was associated with lower costs [5]. 

The strong adsorption of viruses to NanoCeram filters requires an equally robust method of elution. A high-pH solution is commonly used to elute viruses from positively charged filters and adjusted to neutral pH to prevent loss of virus [4,5,6]. Ikner et al. investigated both elution and concentration methods after virus adsorption to a positively charged membrane. Elution using eleven solutions containing variable amounts of beef extract, glycine, phosphate buffer, and sodium polyphosphate were evaluated for viral recovery. The most effective elution was performed using a sodium polyphosphate and glycine buffer solution. The concentrations using Centricon Plus-70 centrifugal filters were also assessed and found to provide similar recovery to reported data using organic flocculation while decreasing the concentrate volume [6]. 

The efficacy of commercial extraction kits in the recovery of enteric viruses was studied by Iker et al. The MO BIO PowerViral Environmental DNA/RNA Isolation kit (now the Qiagen AllPrep PowerViral RNA/DNA kit), QIAamp Viral RNA Mini kit, and the Zymo ZR Virus DNA/RNA Extraction kit were compared. The MO BIO kit allowed for a more consistent recovery of three viruses from three different matrices, including surface water. The MO BIO kit was also the most efficient in the reduction of qPCR inhibitors [7]. 

Recently, the advances in enteric virus recovery and detection from various water types were reviewed by Haramoto et al. The methodologies discussed above were examined as well as the types of controls necessary to analyze a method for an individual experiment. Whole and molecular process controls were employed to examine the efficacy of the entire VIRADEL method or the extraction and detection portions alone. Controls can also be used to monitor inhibition in molecular assays such as RT-qPCR [8].

## 2. Experimental Design

**Figure 1:**
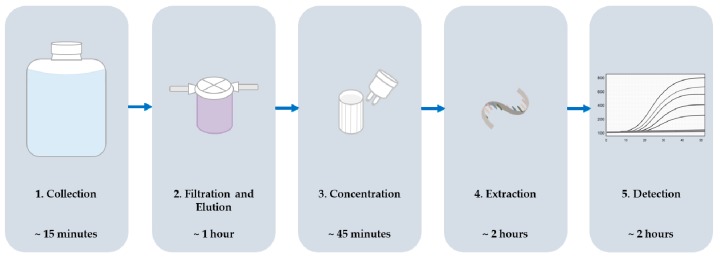
Sample processing scheme including order of steps and the time typically required to complete each step for one sample.

This protocol describes the efficient recovery and detection of enteric viruses from highly variable waters. Surveillance of six Mid-Atlantic U.S. locations (three surface, three reclaimed) was performed over a seventeen-month period with detection for Aichi virus (AiV), hepatitis A virus (HAV), and noroviruses GI and GII (NoV GI and GII). Samples were collected twice monthly, June through September, and once monthly, October through May. A modified VIRADEL method, depicted in Figure 1, was used to recover viruses from water samples and an RT-qPCR assay was used for detection. Viruses were adsorbed to a positively-charged filter, eluted with a high-pH buffer, and concentrated using centrifugal filters. RNA was extracted from the concentrate using a commercially available kit prior to detection. Tulane virus (TV) was used as a molecular control to monitor inhibition during detection. Results were reported as positive or not detected for each virus in all samples.

### 2.1. Materials

#### 2.1.1. Filtration 

• 5-inch NanoCeram Filter (Argonide, Sanford, FL, USA; Cat. no.: VS2.5-5)

#### 2.1.2. Elution and Concentration

- Sodium Phosphate Dibasic (Na_2_HPO_4_) (Millipore Sigma, Burlington, MA, USA; Cat. no.: S9390)
- Potassium Phosphate Monobasic (KH_2_PO_4_) (MP Biomedicals, Solon, OH, USA; Cat. no.: 195453)
- Sodium Polyphosphate (NaPP) (Fisher Scientific, Hampton, NH, USA; Cat. no.: 390932500)
- Glycine (Fisher ScientificCat. no.: G48-500)
- 6N NaOH for pH Adjustment
- 100 kDa Centricon Plus-70 Centrifugal Filters (MilliporeSigma; Cat. no.: UFC710008)

#### 2.1.3. Extraction

- Tulane Virus (a gift from Dr. Xi Jiang, University of Cincinnati College of Medicine, Cincinnati, OH, USA)
- AllPrep PowerViral RNA/DNA Kit (Qiagen, Hilden, Germany; Cat. no.: 28000-50)
- RNase-Free DNase Set (Qiagen; Cat. no.: 79254)

#### 2.1.4. Detection

- tris-EDTA (TE) Buffer (Thermo Fisher Scientific, Waltham, MA, USA; Cat. no.: AM9858)
- QuantiNova Probe RT-PCR Kit (Qiagen; Cat. no.: 208354)
- Strip Tubes and Caps, 0.1 mL (Qiagen; Cat. no.: 981103)
- Custom Probe-Based qPCR Assays, Primers and Probes (Integrated DNA Technologies, Coralville Iowa, USA)
- Norovirus GI synthetic RNA Segment (ATCC, Manassas, VA, USA; Cat. no.: VR-3234SD)
- Norovirus GII synthetic RNA Segment (ATCC; Cat. no.: VR-3235SD)

### 2.2. Equipment

#### 2.2.1. Collection

• Nalgene Autoclavable Carboy (Thermo Fisher Scientfic; Cat. no.: 2250-0050PK)

#### 2.2.2. Filtration and Concentration

- 3.8 GPM FloJet Pump (Xylem, Rye Brook, NY, USA; Cat. no.: 04300042)
- Hydronix Filter Housing Units (iFilters, Ontario, CA, USA; Cat. no.: HF2-5CLWH12)
- Allegra X-12R Centrifuge (Beckman Coulter, Pasadena, CA, USA; Cat. no.: 392302)

#### 2.2.3. Extraction

• Sorvall Legend Micro 21R Centrifuge (Thermo Fisher Scientific; Cat. no.: 75002446)

#### 2.2.4. Detection

• Roto-Gene Q 2plex Platform Apparatus (Qiagen; Cat. no.: 9001550)

## 3. Procedure

Below is the protocol for the recovery and detection of enteric viruses (AiV, HAV, NoV GI, and NoV GII) from non-traditional irrigation water sources.

### 3.1. Sample Collection. Time for Completion: 00:15 Minutes

1. Collect water samples using sterile 20 liter carboys. Submerge the carboy below the water surface, without disrupting sediment, use a pump to pump water into the carboy, or place beneath a tap and allow the carboy to fill.
2. Transport samples to the laboratory and process immediately or refrigerate for up to 24 h.

### 3.2. Filtration and Elution. Time for Completion: 01:00 H

**Figure 2:**
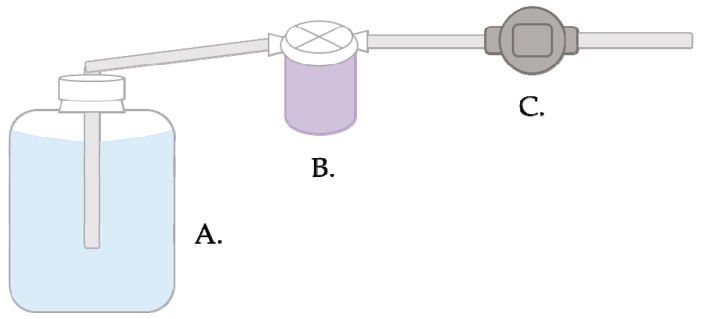
Filtration setup and water flow diagram. One end of tubing is placed in the water sample (**A**) and connected on the other end to the filter housing unit (**B**). Water is pulled through the filter in the housing unit and another piece of connected tubing by the pump at a maximum flow rate of 8.5 L/minute (**C**). Water is discarded through the final piece of tubing.

1. Place a new sterile unused filter into a housing unit and connect the tubing (as seen in Figure 2)
2. Connect two pieces of tubing to the filter housing unit and insert one piece of tubing into the carboy and attach the other to the pump.
3. Turn on the pump and allow the water to flow through the filter until filtration is complete.
  i **OPTIONAL STEP** More than one filter may be used in this step if the filter becomes clogged.
4. Disconnect the tubing and perform elution or freeze filters for up to 24 hours.
5. Continue with the elution or repeat Steps 1–4 for additional samples.
  a) 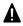 **CRITICAL STEP** New tubing should be used (between A and B in Figure 1) or previously used tubing should be bleached to reduce to risk of contamination between samples.
6. Add 300 mL of sodium polyphosphate (NaPP) buffer to each housing unit and invert the unit 10 times.
7. Allow the unit to incubate at room temperature for 15 min.
8. Invert the unit 10 times and transfer the eluate to a sterile container.
9. Transfer the eluate to the housing unit to rinse the filter and again to the container.
10. Adjust the pH of eluates to 7.2 ± 0.2 using NaOH.
  **CRITICAL STEP** Add NaOH slowly to avoid loss of virus and decreasing the pH below the desired range.
11. Concentrate the eluate immediately or refrigerate for up to 24 h.

### 3.3. Concentration. Time for Completion: 00:45 Min

1. Add 60 mL of eluate to the top compartment of each centrifugal filter. Four filters are used to concentrate up to 240 mL of eluate. The remainder of eluate is stored at −80 °C or additional filters may be used to concentrate the entire volume.
2. Place the lid securely on the filter and centrifuge at 1900 *x g* for 8 min.
3. Remove the filtrate from the bottom compartment
  a) **OPTIONAL STEP** If all of the 60 mL does not pass through the filter, an additional filter may be used.
4. Repeat Steps 1–3 using sterile water in the top compartment of each filter.
5. Invert the filter and centrifuge at 800 *x g* for 2 min.
6. Pool the virus concentrate and store at −80 °C until performing extractions. The volume of concentrate varies between 500 µL and 14,500 µL.

### 3.4. Extraction and DNA removal. Time for Completion: 02:00 H

1. If frozen, remove the concentrate and allow to thaw at room temperature.
2. Centrifuge the thawed concentrate for 1 minute at 12,000 *x g*.
3. Remove 200 µL of the supernatant and add 4 µL of TV, 5 log(copies/reaction), to each sample.
4. Perform extractions in duplicate using Qiagen’s AllPrep PowerViral kit according to the manufacturer’s instructions.
  - Do not perform optional Steps 3–7 of Qiagen protocol for bead beating.
  - Between Steps 13 and 14 of the Qiagen protocol perform the DNase protocol according to the manufacturer’s instructions.

### 3.5. Detection via RT-qPCR Assay. Time for Completion: 02:00 H

1. Concentrations of primers and probes should be adjusted to a working concentration prior to combining reagents (Table 1) and each set added to the appropriate reaction mix.
2. RT-qPCR reagents are prepared and combined according to the manufacturer’s instructions (Table 2).
3. Perform detection for each sample in triplicate and include positive and negative controls for each RT-qPCR run.
4. The qPCR thermocycler was programmed as follows: 45 °C for 10 min, 95 °C for 5 min, and fifty cycles of 95 °C for 5 seconds and 60 °C for 30 s.

## 4. Expected Results

- No samples exhibited inhibition during detection using this protocol.
- If RT-qPCR is performed using a standard curve and the positive results are detected above the limit of detection, a comparison of detected levels may be performed. In this study, results were reported only as positive or not detected (ND).
- Concentrated viruses were spiked with the TV control prior to extraction. For a more comprehensive idea of virus recovery, TV could be introduced prior to filtration. This will not allow for absolute quantification as all viruses vary structurally. This study utilized TV as a control for successful extraction and detection of nucleic acids only.

## 5. Reagents Setup

- NaPP buffer is prepared by combining 0.54 g/L Na_2_HPO_4_, 0.88 g/L KH_2_PO_4_, 3.75 g/L glycine, and 10 g/L NaPP. The final concentrations of reagents are 0.0038M Na_2_HPO_4_, 0.0065M KH_2_PO_4_, 0.050M glycine, and 0.027M NaPP. The buffer requires autoclaving and cooling prior to pH adjustment to 9.3 using 6N NaOH.
- Extraction kit and DNase reagents were prepared and stored according to the manufacturer’s instructions [13,14].
- Custom primers and probes were reconstituted and diluted to the concentrations (described in the respective manuscripts) according to the manufacturer’s instructions [15,16].

## Figures and Tables

**Table 1:** Aichi virus (AiV), hepatitis A virus (HAV), noroviruses GI and GII (NoV GI and GII) custom primers and probes sequences and concentrations used for the detection of viral targets.

Target	Primer Set	Sequence (5′-3′)	Stock Concentration (nM)	Reaction Concentration (nM)	Reference
AiV	Forward	GTCTCCACHGACACYAAYTGGAC	8000	400	[9]
	Reverse	GTTGTACATRGCAGCCCAGG	8000	400	
	Probe	FAM-TTYTCCTTYGTGCGTGC-BHQ1	6000	300	
HAV	Forward	GGTAGGCTACGGGTGAAAC	5000	250	[10]
	Reverse	AACAACTCACCAATATCCGC	5000	250	
	Probe	FAM-CTTAGGCTAATACTTCTATGAAGAGATGC-BHQ1	3000	150	
NoV GI	Forward	CGYTGGATGCGNTTYCATGA	8000	400	[11]
	Reverse	CTTAGACGCCATCATCATTYAC	8000	400	
	Probe 1	FAM-AGATYGCGATCYCCTGTCCA-BHQ1	6000	300	
	Probe 2	FAM-AGATCGCGGTCTCCTGTCCA-BHQ1	2000	100	
NoV GII	Forward	CARGARBCNATGTTYAGRTGGATGAG	8000	400	[11]
	Reverse	TCGACGCCATCTTCATTCACA	8000	400	
	Probe	FAM-TGGGAGGGCGATCGCAATCT-BHQ1	6000	300	
TV	Forward	GACGATGACCTTGCGTG	6000	300	[12]
	Reverse	TGGGATTCAACCATGATACAGTC	6000	300	
	Probe	FAM-ACCCCAAAGCCCCAGAGTTGAT-BHQ1	2000	100	

**Table 2:** Volumes of reagents added to each RT-qPCR reaction.

Reagent	Volume (µL)
Master Mix	10.0
RT Mix	0.2
Forward Primer	1.0
Reverse Primer	1.0
Probe *	1.0
Template RNA **	2.0
RNase-Free Water	Balance
Total	20

* NoV GII requires two probes, each should be added in 1 µL volumes. ** Template RNA can be replaced with synthetic gBlock and RNase-free water for positive and negative controls.
